# Clinical characteristics and outcome of iatrogenic colonic perforation related to diagnostic vs. therapeutic colonoscopy

**DOI:** 10.1007/s00464-022-09010-6

**Published:** 2022-01-19

**Authors:** Ra Ri Cha, Hee Jin Kim, Chang Min Lee, Jae Min Lee, Sang Soo Lee, Hyun Jin Cho, Chang Yoon Ha, Hyun Jin Kim, Ok-Jae Lee

**Affiliations:** 1 Department of Internal Medicine, Gyeongsang National University College of Medicine, 816 beon-gil 15, Jinu-daero, Jinju, Gyeongnam 52727 Republic of Korea; 2 Division of Gastroenterology, Gyeongsang National University Changwon Hospital, Changwon, Republic of Korea; 3 Division of Gastroenterology, Gyeongsang National University Hospital, Jinju, Republic of Korea; 4 Gyeongsang National University Institute of Health Sciences, Jinju, Republic of Korea

**Keywords:** Colonoscopy, Colonic perforation, Iatrogenic, Endoscopic treatment

## Abstract

**Aim:**

Iatrogenic colonic perforation (ICP) is a rare serious complication of colonoscopy, where standard treatment is controversial. This study aimed to characterize diagnostic ICP (DICP) compared to therapeutic ICP (TICP) and determine the possible indication of endoscopic repair.

**Methods:**

We studied patients with ICP over 7 years starting in 2011. Their demographics and data regarding perforation, treatment, and outcome were investigated by retrospective review of medical records, and the diagnostic and therapeutic groups were compared.

**Results:**

Among 29,882 patients who underwent colonoscopy, ICP was identified in 28 (0.09%: diagnostic, 15/24,758, 0.06%; therapeutic, 13/5124, 0.25%). A total of 56 patients (33 DICP and 23 TICP) including 28 referred cases were analyzed. Mean age was 62.3 ± 11.4 years, and 24 were men. Perforations occurred mostly in the rectosigmoid region and half were detected during or immediately after colonoscopy. Endoscopic treatment was successful in 22 cases and 34 required surgery. Mortality occurred in 4 (7.1%).

Compared to TICP, DICP was more prevalent in females and rectosigmoid region and more frequently detected immediately (all *p* < 0.05); DICP tended to occur in older patients, be larger and have better chance of endoscopic repair.

Regardless of type of ICP, female predominance, smaller perforation, more frequent immediate detection, and shorter hospital stay (all *p* = 0.01) were found in the endoscopic repair group.

**Conclusion:**

DICP was more frequent in the rectosigmoid area in older women and could be detected immediately. Immediate detection and small perforation size could be important factors for endoscopic repair. Careful attention and gentle manipulation should be required.

Colonoscopy is a relatively safe diagnostic or therapeutic tool for colorectal disease. However, with the increasing number of procedures performed, colonic perforation is an almost unavoidable, well-recognized complication of diagnostic or therapeutic colonoscopy. The frequency of colonic perforation during diagnostic and therapeutic colonoscopy has been reported to be 0.1 ~ 0.8% and 0.15 ~ 3% [1–3], respectively, and the risk increases to 1.9% in elderly patients [4]. Increasing use of more invasive therapeutic procedures such as endoscopic mucosal resection (EMR) or endoscopic submucosal dissection (ESD) increase the risk of complications.

Despite the rarity of colonic perforation, it is associated with a high rate of morbidity and mortality. This complication could lead to operation, stoma formation, intra-abdominal sepsis, prolonged hospital stay, and even death [2, 5, 6]. The standard treatment for iatrogenic colonic perforation (ICP) is controversial, because no randomized study has ever been conducted. Furthermore, the indication, efficacy, and complications of endoscopic treatment compared to surgical treatment have not been fully elucidated.

The purpose of this study was to assess the frequency, clinical characteristics, and management and outcome of ICP related to diagnostic (DICP) compared to therapeutic (TICP) colonoscopy, and to determine the possible indication of endoscopic repair.

## Materials and methods

### Patients

We studied patients who were admitted to our tertiary Gyeongsang National University Hospital for management of ICP that occurred during diagnostic or therapeutic colonoscopy from January 2011 to December 2017. Cases referred from secondary or primary clinics were also included. Informed consent was obtained from all study subjects.

The study protocol was approved by the Institutional Review Board of Gyeongsang National University Hospital (IRB Number: 2019-07-022).

### Methods

We reviewed retrospectively the medical records of the study patients with ICP. The patients were classified into two groups; the diagnostic and therapeutic ICP groups, DICP and TICP, respectively. We investigated the patients’ demographic data, comorbidity, prior abdominal surgery, indication of colonoscopy, procedure performed, and the data regarding perforation including location, size, presenting symptom, treatment and outcome, and compared the data of the two groups. Additionally, we analyzed the characteristics of the successful endoscopic repair group compared to the surgical treatment group.

Colonoscopy was classified as diagnostic when mucosal biopsy with retrieval of specimen using forceps was the only procedure performed, and it was therapeutic when an endoluminal procedure such as polypectomy, EMR, or ESD was carried out.

Colonic perforation was diagnosed by direct visualization of extraintestinal structures during colonoscopy, presence of pneumoperitoneum or retroperitoneal gas with signs of peritonitis after the procedure, and intraoperative findings of a perforated colon. The perforation size was obtained from the endoscopic and/or surgical records. Endoscopic repair indicated closure of perforation by hemoclipping or ligation with an elastic band, while surgical repair included simple closure or colonic resection with or without ileostomy via laparoscopy or laparotomy. Once perforation was recognized, immediate intravenous fluids and broad spectrum antibiotics were administered concurrently with endoscopic or surgical repair in all patients with ICP.

### Statistical analysis

Statistical analysis was performed using SPSS version 19.0.0 (IBM Co., Armonk, NY, USA). The *t *test, Fisher’s exact test, and chi-square test were performed as appropriate. *p* values less than 0.05 were considered statistically significant.

## Results

### Frequency of iatrogenic colonic perforation

**Fig. 1:**
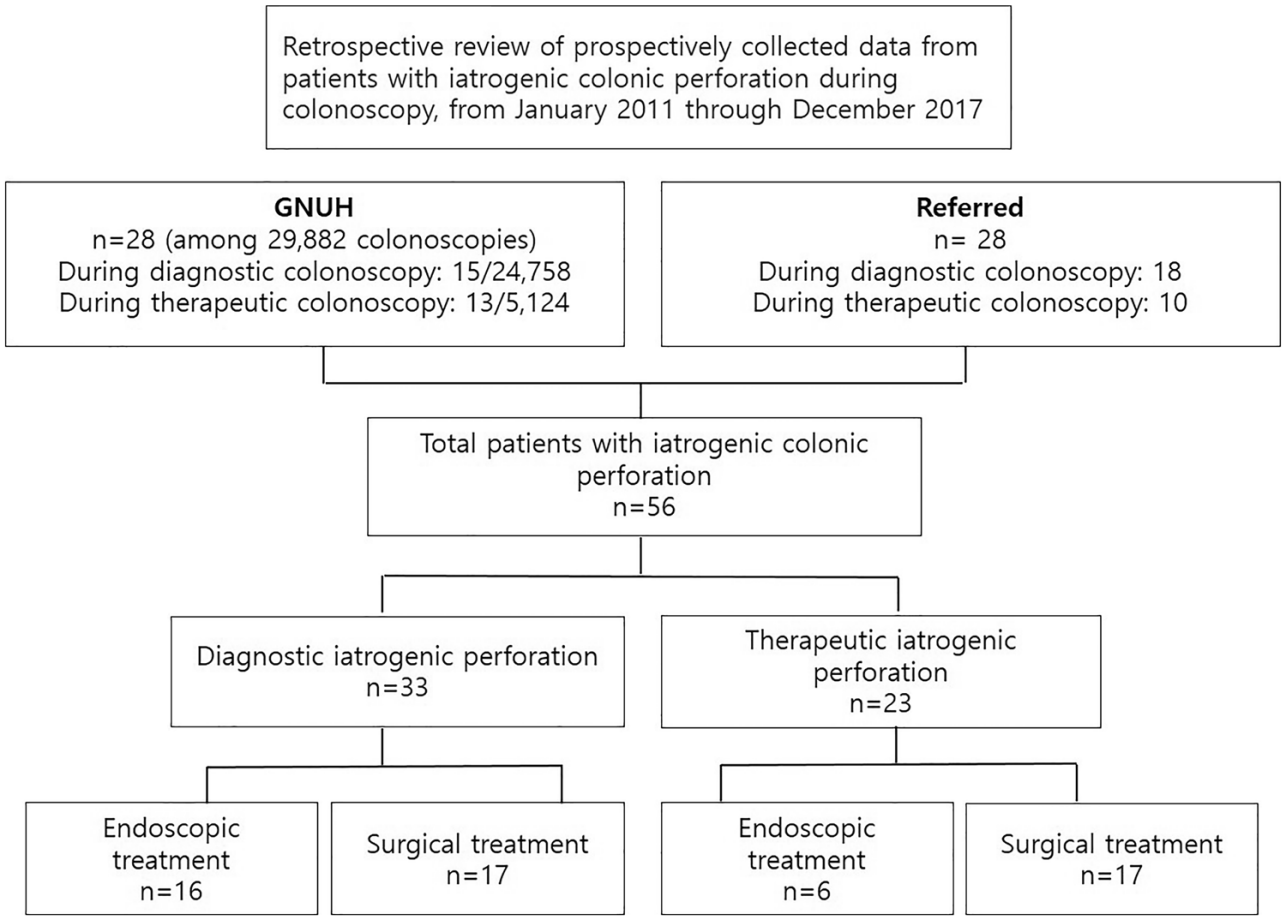
Schematic flowchart of the patients with iatrogenic colonic perforation during diagnostic or therapeutic colonoscopy. *GNUH* Gyeongsang National University Hospital

A total of 56 cases of ICP were identified during 7 years starting January 2011 and 33 of these were related to diagnostic colonoscopy and the other 23 were related to therapeutic procedures. Actually, 28 cases of colonic perforation occurred in 29,882 colonoscopic procedures (24,758 diagnostic and 5124 therapeutic) performed at Gyeongsang National University Hospital (tertiary center) during the same period, and the other 28 were referred from a primary or secondary center (Fig. 1). Therefore, the overall frequency of ICP was 0.09% (28/29,882), and diagnostic and therapeutic colonoscopy-related perforation rates were 0.06% (15/24,758) and 0.25% (13/5124), respectively (Table 1).

**Table 1:** Number of colonoscopies and the frequency of iatrogenic colonic perforation at Gyeongsang National University Hospital (tertiary hospital)

Year	Number of cases			Number of perforations (%)		
	Diagnostic	Therapeutic	Total	Diagnostic	Therapeutic	Total
2011	3570	624	4194	0 (0.00%)	2 (0.32%)	2 (0.05%)
2012	3610	503	4113	4 (0.12%)	1 (0.20%)	5 (0.12%)
2013	3458	567	4025	1 (0.03%)	2 (0.35%)	3 (0.07%)
2014	3615	690	4305	3 (0.08%)	2 (0.29%)	5 (0.12%)
2015	3639	804	4443	4 (0.10%)	3 (0.37%)	7 (0.16%)
2016	3326	1116	4442	2 (0.06%)	2 (0.18%)	4 (0.09%)
2017	3540	820	4360	1 (0.03%)	1 (0.12%)	2 (0.05%)
Total	24,758	5124	29,882	15 (0.06%)	13 (0.25%)	28 (0.09%)

### Clinical characteristics of iatrogenic perforation; DICP vs. TICP

The patients’ mean age was 62.3 ± 11.4 years and 30 (53.6%) were males. Among 56 cases with ICP including the referred cases, 24 (42.9%) had comorbidity and 12 (21.4%) had history of abdominal surgery. Indication for colonoscopy was screening purpose (*n* = 34, 60.7%), evaluation for gastrointestinal symptoms (*n* = 9, 16.1%), and endoscopic removal of neoplasm such as polypectomy, EMR, or ESD (*n* = 13, 23.2%). The most common clinical presentation of perforation was abdominal pain (*n* = 45, 80.4%), followed by fever, abdominal distension, and bleeding, whereas 4 (7.1%) patients did not have any symptoms. The most common site of colonic perforation was the rectosigmoid area (*n* = 36, 64.3%), followed by ascending colon (*n* = 9, 16.1%), transverse colon (*n* = 5, 8.9%), descending colon (*n* = 5, 8.9%), and cecum (*n* = 1, 1.8%). The median perforation size was 1.00 cm (0.3–7.0 cm). The colonic perforation was detected during or immediately after the completion of procedure in 28 (50.0%) cases, and within 24 h in 15 (26.8%), while 13 (23.2%) cases were identified after 24 h. Endoscopic treatment was successful in 22 cases (39.4%) without mortality, but 34 cases (60.7%) required surgical repair. The median fasting time was 6.0 days (2–30 days), and the median hospital stay was 13.6 days (2–71 days). 52 (92.9%) patients recovered, but 4 (7.1%) patients died of colonic perforation-related or other causes after surgical treatment.

There were 33 (58.9%) cases of DICP and 23 (41.1%) of TICP. Compared to the TICP group, the DICP group was older (72.8 ± 8.3 vs. 65.2 ± 2.7 years, *p* = 0.080) with female predominance (52.6 vs. 30.4%, *p* = 0.045). Presence of comorbidity (51.5 vs. 69.6%, *p* = 0.177) and history of prior abdominal surgery (18.2 vs. 26.1%, *p* = 0.177) was similar in the two groups. There were no significant differences in presenting symptom of colonic perforation (*p* = 0.450). Abdominal pain was the most common symptom in both groups. DICP developed most frequently in the rectosigmoid region (84.8%, 28/33), while TICP occurred in the colon anywhere the procedure was performed (*p* = 0.000). The size of perforation was relatively larger in the DICP than TICP group but without statistical significance (1.21 vs. 0.91 cm, *p* = 0.080). In all DICP cases except one, perforation was detected immediately (24, 72.7%) or within 24 h (8, 24.2%). However, in over half of patients in the therapeutic group, there was a delay of over 24 h in identifying the perforation (12, 52.2%) (*p* = 0.000). Endoscopic repair was successful in 48.5% of the diagnostic group and 26.1% of the therapeutic group; meanwhile, surgical repair was required in 51.5% of the diagnostic group and 73.9% of the therapeutic group (*p* = 0.091). Three DICP and one TICP patients died (9.1 vs. 4.3%, *p* = 0.498). There were no significant differences in the fasting time (*p* = 0.953) and length of hospital stay (*p* = 0.332) between the two groups (Table 2).

**Table 2:** Clinical characteristics and outcome of patients with iatrogenic colonic perforation during diagnostic or therapeutic colonoscopy

Variable	Total *n* = 56	Diagnostic *n* = 33 (58.9%)	Therapeutic *n* = 23 (41.1%)	*p* value
Age (years, mean ± SD)	62.3 ± 11.4	72.8 ± 8.3	65.2 ± 2.7	0.080
Gender				0.045
Male	30 (53.6%)	14 (42.4%)	16 (69.6%)	
Female	26 (46.4%)	19 (52.6%)	7 (30.4%)	
Hospital of occurrence				0.415
GNUH (tertiary)	28 (50.0%)	15 (45.5%)	13 (56.5%)	
Referred (primary or secondary)	28 (50.0%)	18 (54.5%)	10 (43.5%)	
Comorbidity				0.177
Presence	33 (58.9%)	17 (51.5%)	16 (69.6%)	
Absence	23 (41.1%)	16 (48.5%)	7 (30.4%)	
BMI (mean ± SD)	21.9 ± 2.14	21.5 ± 2.11	22.5 ± 2.08	0.862
Prior abdominal surgery				0.478
Yes	12 (21.4%)	6 (18.2%)	6 (26.1%)	
No	44 (78.6%)	27 (81.8%)	17 (73.9%)	
Indication of colonoscopy				0.000
Screening	34 (60.7%)	25 (75.8%)	9 (39.1.3%)	
Gastrointestinal symptoms	9 (16.1%)	8 (24.2%)	1 (4.3%)	
Endoscopic removal	13 (23.2%)	0 (0.0%)	13 (56.5%)	
Clinical presentation				0.450
No symptom	4 (7.1%)	1 (3.0%)	3 (13.0%)	
Abdominal pain	45 (80.4%)	28 (84.8%)	17 (73.9%)	
Abdominal distension	3 (5.4%)	2 (6.1%)	1 (4.3%)	
Fever	3 (5.4%)	2 (6.1%)	1 (4.3%)	
Bleeding	1 (1.8%)	0 (0.0%)	1 (4.3%)	
Site of perforation				0.000
Rectosigmoid	36 (64.3%)	28 (84.8%)	8 (34.8%)	
Descending colon	5 (8.9%)	3 (9.1%)	2 (8.7%)	
Transverse colon	5 (8.9%)	2 (6.1%)	3 (13.0%)	
Ascending colon	9 (16.1%)	0 (0.0%)	9 (39.1%)	
Cecum	1 (1.8%)	0 (0.0%)	1 (4.3%)	
Size of perforation [cm, median (range)]	1.00 (0.3–7.0)	1.21 (0.3–7.0)	0.91(0.5–2.0)	0.080
Time of diagnosis				0.000
During examination or immediate	28 (50.0%)	24 (72.7%)	4 (17.4%)	
< 24 h	15 (26.8%)	8 (24.2%)	7 (30.4%)	
≥ 24 h	13 (23.2%)	1 (3.0%)	12 (52.2%)	
Management of treatment				0.091
Endoscopic treatment	22 (39.3%)	16 (48.5%)	6 (26.1%)	
Surgical treatment	34 (60.7%)	17 (51.5%)	17 (73.9%)	
Outcome				0.498
Recovery	52 (92.9%)	30 (90.9%)	22 (95.7%)	
Death	4 (7.1%)	3 (9.1%)	1 (4.3%)	
Fasting time [days, median (range)]	6.0 (2–30)	6.0 (2–19)	5.8 (3–30)	0.953
Hospital stay [days, median (range)]	13.6 (2–71)	14.0 (2–71)	12.0 (5–36)	0.332

*SD* standard deviation; *GNUH* Gyeongsang National University Hospital; *BMI* body mass index

### Comparison of clinical characteristics of iatrogenic colonic perforation according to treatment option; endoscopic vs surgical treatment

Compared to the surgical treatment group, the endoscopic repair group was predominantly female (68.2 vs. 32.4%, *p* = 0.009). Perforation size was smaller (0.81 vs. 1.30 cm, *p* = 0.001), and immediate detection was more common (86.45 vs. 26.5%, *p* = 0.000) in the endoscopic repair group than surgical treatment group. Meanwhile delayed detection over 24 h was more frequent in the surgical treatment group than endoscopic repair group (35.3 vs. 4.5%). All in the endoscopic repair group recovered without complication (100%), 4 cases in the surgical treatment group died (11.8%). Fasting time was shorter in the endoscopic repair than surgical treatment group but without statistical significance (5.3 vs. 6.5 days, *p* = 0.090), but hospital stay was significantly shorter in the endoscopic repair group (8.6 vs. 15.8 days, *p* = 0.007). There was no significant difference in patients’ age (69.3 ± 11.20 vs. 65.9 ± 11.51 years, *p* = 0.284), center of occurrence (*p* = 0.274), comorbidity (*p* = 0.258), history of prior abdominal surgery (*p* = 0.391), indication of colonoscopy (*p* = 0.910), clinical presentation (*p* = 0.919), and site of perforation (*p* = 0.510) between the two groups (Table 3).

**Table 3:** Clinical characteristics and outcome of patients with iatrogenic colonic perforation according to treatment of perforation

Variable	Endoscopic treatment			Surgical treatment			*p* value
	Total (*n* = 22)	Diagnostic (*n* = 16)	Therapeutic (*n* = 6)	Total (*n* = 34)	Diagnostic (*n* = 17)	Therapeutic (*n* = 17)	
Age (years, mean ± SD)	69.3 ± 11.20	70.4 ± 10.08	66.3 ± 14.40	65.9 ± 11.51	70.4 ± 7.82	61.4 ± 13.02	0.284
Gender							0.009
Male	7 (31.8%)	4 (25.0%)	3 (50.0%)	23 (67.6%)	10 (58.8%)	13 (76.5%)	
Female	15 (68.2%)	12 (75.0%)	3 (50.0%)	11 (32.4%)	7 (41.2%)	4 (23.5%)	
Hospital of occurrence							0.274
GNUH (tertiary)	13 (59.1%)	9 (56.2%)	4 (66.7%)	15 (44.1%)	6 (35.3%)	9 (52.9%)	
Referred (primary or secondary)	9 (40.9%)	7 (43.8%)	2 (33.3%)	19 (55.9%)	11 (64.7%)	8 (47.1%)	
Comorbidity							0.258
Presence	15 (68.2%)	10 (62.5%)	5 (83.3%)	18 (52.9%)	7 (41.2%)	11 (64.7%)	
Absence	7 (31.8%)	6 (37.5%)	1 (16.7%)	16 (47.1%)	10 (58.8%)	6 (35.3%)	
Prior abdominal surgery							0.391
Yes	16 (72.7%)	12 (75.0%)	4 (66.7%)	28 (82.4%)	15 (88.2%)	13 (76.5%)	
No	6 (27.3%)	4 (25.0%)	2 (33.3%)	6 (17.6%)	2 (11.8%)	4 (23.5%)	
Indication of colonoscopy							0.910
Screening	14 (63.6%)	13 (81.2%)	1 (16.7%)	20 (58.8%)	12 (70.6%)	8(47.1%)	
Gastrointestinal symptom	3 (13.6%)	3 (18.8%)	0 (0.0%)	6 (17.6%)	5 (29.4%)	1(5.9%)	
Endoscopic removal	5 (22.7%)	0 (0.0%)	5(83.3%)	8 (23.5%)	0(0.0%)	8(47.1%)	
Clinical presentation							0.919
No symptom	2 (9.1%)	1 (6.2%)	1 (16.7%)	2 (5.9%)	0 (0.0%)	2 (11.8%)	
Abdominal pain	18 (81.8%)	13 (81.2%)	5 (83.3%)	27 (79.4%)	15 (88.2%)	12 (70.6%)	
Abdominal distension	1 (4.5%)	1 (6.2%)	0 (0.0%)	2 (5.9%)	1(5.9%)	1 (5.9%)	
Bleeding	0 (0.0%)	0 (0.0%)	0 (0.0%)	1 (2.9%)	0 (0.0%)	1 (5.9%)	
Fever	1 (4.5%)	1 (6.2%)	0 (0.0%)	2 (5.9%)	1 (5.9%)	1 (5.9%)	
Site of Perforation							0.510
Rectosigmoid	15 (68.2%)	15 (93.8%)	0 (0.0%)	21 (61.8%)	13 (76.5%)	8 (47.1%)	
Descending colon	1 (4.5%)	1 (6.2%)	0 (0.0%)	4 (11.8%)	2 (11.8%)	2 (11.8%)	
Transverse colon	1 (4.5%)	0 (0.0%)	1 (16.7%)	4 (11.8%)	2 (11.8%)	2 (11.8%)	
Ascending colon	5 (22.7%)	0 (0.0%)	5 (83.3%)	4 (11.8%)	0 (0.0%)	4 (23.5%)	
Cecum	0 (0.0%)	0 (0.0%)	0 (0.0%)	1 (2.9%)	0 (0.0%)	1 (5.9%)	
Size of perforation [cm, median (range)]	0.81 (0.3–1.0)	0.81 (0.3–1.0)	0.58 (0.5–1.0)	1.30 (0.5–7.0)	1.70 (1.0–7.0)	1.09 (0.5–2.0)	0.001
Time of detection							0.000
Immediate	19 (86.4%)	15 (93.8%)	4 (66.7%)	9 (26.5%)	9 (52.9%)	0 (0.0%)	
< 24 h	2 (9.1%)	1 (6.2%)	1 (16.7%)	13 (38.2%)	7 (41.2%)	6 (35.3%)	
≥ 24 h	1 (4.5%)	0 (0.0%)	1 (16.7%)	12 (35.3%)	1 (5.9%)	11 (64.7%)	
Outcome							0.095
Recovery	22 (100.0%)	16 (100.0%)	6 (100.0%)	30 (88.2%)	14 (82.4%)	16 (94.1%)	
Death	0 (0.0%)	0 (0.0%)	0 (0.0%)	4 (11.8%)	3 (17.6%)	1 (5.9%)	
Fasting time [days, median (range)]	5.3 (2–14)	5.3 (2–14)	5.3 (3–9)	6.5 (3–30)	7.0 (3–19)	6.1 (4–30)	0.090
Hospital stay [days, median (range)]	8.6 (3–30)	9.3 (3–30)	8.0 (5–18)	15.8 (2–71)	19.0 (2–71)	14.5 (8–36)	0.007

*SD* standard deviation; *GNUH* Gyeongsang National University Hospital

### Clinical characteristics of mortality cases

Four patients (7.1%) died; 3 (9.1%) were in diagnostic group and the other one (4.3%) in the therapeutic group. Three were males and all patients were over 60 years old. The comorbidities in each of the four patients are shown in Table 4. ICP was not identified during or immediately after study in all mortality cases; that is, 3 were detected within 24 h, and the other one was identified after 24 h. The site of colonic perforation had no character in common; 2 were sigmoid colon and the others transverse and ascending colon. The size of colonic perforation varied but over 1 cm in all cases (1 ~ 2.5 cm). All patients with ICP required surgical treatment. One patient died from postoperative sepsis, and three patients died from multiple-organ failure.

**Table 4:** Detailed summary of the 4 patients who died

Sex	Age	Comorbidity	Type/institution	Site of perforation	Size of perforation	Time to detect	Treatment	Cause of death	Hospital stay
M	66	CVA, COPD, DM	Diagnostic/GNUH	Sigmoid colon	2.5 cm	< 24 h	Ileostomy with wedge resection	Sepsis	63 days
F	64	LC, angina	Diagnostic/GNUH	Transverse colon	1.5 cm	≥ 24 h	Ileostomy with wedge resection	Multi-organ failure	44 days
M	60	LC	Therapeutic/referred	Ascending colon	1.5 cm	< 24 h	Right hemicolectomy	Multi-organ failure	2 days
M	70	DM, HT	Diagnostic/GNUH	Sigmoid colon	1 cm	< 24 h	Primary repair	Multi-organ failure	25 days

*M* male; *F* female; *CVA* cerebrovascular accident; *COPD* chronic obstructive pulmonary disease; *DM* diabetes mellitus; *LC* liver cirrhosis; *HT* hypertension; *GNUH* Gyeongsang National University Hospital

## Discussion

In this retrospective study, we found that ICP developed in 0.09% of the subjects who had undergone diagnostic or therapeutic colonoscopy during 7 years, and the rate of DICP was 0.06%. DICP mostly occurred in the rectosigmoid area and it was more frequent in women, especially older ones, compared to TICP. Fortunately, it could be identified immediately, and there was a chance of endoscopic repair, avoiding surgery. In particular, the perforation size was significantly smaller (0.8 cm, 0.3–1.0 cm) and immediate detection was more common in the successful endoscopic repair group compared to surgical treatment group, regardless of type of ICP. These findings suggest that smaller size and immediate detection of perforation could be important for indication of endoscopic repair.

Regarding the ICP rate, our results are comparable to those of previous studies in high-volume centers, with estimations of between 0.01 and 0.6% [7, 8]. This wide variation is explained by differences in the proficiency of endoscopist and how many total or difficult therapeutic procedures are performed. Actually, our data also showed variations in annual perforation rate from 0.05 to 0.16%, depending on the number of therapeutic colonoscopies. The risk of perforation in therapeutic colonoscopy is usually higher than that in diagnostic colonoscopy. Excluding TICP, the overall DICP rate was 0.06%, lower than the 0.25% rate of therapeutic colonic perforation. In addition, the variation may be explained by how reliably the medical records and centers were managed and searched for data on ICPs. Moreover, some small perforations can be missed completely, remain subclinical, and heal spontaneously. We, however, collected the consecutive cases with ICP retrospectively so that the patients’ medical records were reviewed thoroughly. Even though subclinical colonic microperforation could be missed, it would not be significant clinically.

There are probably different mechanisms in perforation between diagnostic colonoscopy and therapeutic colonoscopy. Perforations associated with diagnostic colonoscopy most often result from pressure on the colonic wall or excessive air insufflation [9]. The tip of the endoscope can directly cause mechanical damage, and also, forceful stretching of the bowel, especially when loops are formed, causes the mechanical tear of the bowel wall. If there is a narrow lumen, due to stenosis or obstruction, too competent an ileocecal valve, and prolonged procedure with poor bowel preparation and difficult introduction of the endoscope, trapping a large amount of air in an isolated segment of the colon can be dangerous [9]. On the other hand, in therapeutic procedures, ischemia of the colonic wall caused by electrical or thermal injury after electrocoagulation results in perforation. Thus, most DICPs can be diagnosed immediately during the procedure due to visualization of the perforation site and extraintestinal fat or vessels by the endoscopist. Meanwhile, perforations after therapeutic procedures are often diagnosed late because they present a few hours or days later with symptoms. Our results also showed that most patients with DICP (72.7%) were detected immediately, compared to TICP, in which diagnosid was immediate in only 17.4% but in most cases delayed after the procedure (*p* = 0.000, Table 2).

A likely reason for immediate detection of DICP is that diagnostic perforations have been found to produce a larger defect than therapeutic ones (mean size of 1.9 ~ 3.3 vs. 0.9 ~ 1.5 cm, respectively) [10, 11]. This difference may also be explained by the different mechanism of perforation in diagnostic and therapeutic colonoscopies. In our study, the size of perforation was larger in DICP than TICP, but the difference was not statistically significant (1.21 vs. 0.91, *p* = 0.080, Table 2). Our patients with ICP were elderly (62.3 ± 11.4 years), and moreover, DICP patients tended to be older and mostly females, compared to TICP patients. In the elderly, colonic wall mechanical strength might have declined partly as a consequence of changes in collagen structure [12]. Perhaps increased diverticular disease in the elderly may contribute to a higher rate of ICP because an endoscopist could inadvertently push a scope through a large diverticulum, or snare an inverted diverticulum simulating a polyp [13]. In this study, we also had one case of diverticular perforation occurred during diagnostic colonoscopy in an 84 years old female patient with left colonic diverticulosis. After colonoscopy, abdominal pain and distention were noted and ICP was recognized by simple abdominal x rays. Laparoscopy was performed immediately, and the diverticular perforation was identified and treated with laparoscopic wedge resection. She was discharged without sequela one week after surgery.

In this study, the most frequent site of DICPs was the rectosigmoid region, compared to TICPs, which occurred in any segment of the entire colon, especially 67% other than the rectosigmoid segment (84.8 vs. 34.8%, *p* = 0.000, Table 2). The anatomical characteristics of the rectosigmoid colon may explain its vulnerability to mechanical damage during colonoscopy. Since the rectosigmoid colon has redundancy and sharp angulation [14], the sinuous sigmoid segment is the most difficult portion to negotiate with the colonoscope [15]. Additionally, the sigmoid colon is the narrowest portion in the large intestine. The caliber of the colon gradually diminishes distally, from a maximal diameter at the cecum (about 8.5 cm) to a minimal diameter in the sigmoid segment (about 2.5 cm) [15].

Retroflexion was the main cause of perforations in the rectum, while perforation in the sigmoid and rest of colon was attributed to excessive pushing of the colonoscopic tip [16]. Thus, the endoscopist, especially beginner, should be careful to avoid iatrogenic perforation of the rectosigmoid segment during colonoscopy.

ICP can also occur because of adhesions after previous abdominal operations [14]. Abdominal surgery can be accompanied by postoperative adhesion, which can be an important cause of perforation during diagnostic or therapeutic colonoscopy. Here, 12 cases (21.4%) had a previous abdominal operation, and its frequency was higher in the TICP than DICP group but without statistical significance (18.2 vs. 26.1%, *p* = 0.478, Table 2). Therefore, care is needed during colonoscopy regardless of purpose in those subjects who had prior abdominal surgery.

The management of ICP may be endoscopic or surgical and should be selective. The decision on endoscopic or surgical treatment depends on the possible mechanism and size of the perforation, timing of diagnosis, quality of bowel preparation, clinical stability of patients, underlying colonic pathology, and endoscopist’s expertise [17–23]. Traditionally, ICPs have been managed by surgical treatment, but endoscopic closure may result in better outcomes than those of surgical treatment in appropriately selected cases. Recent studies have demonstrated the possibility of endoscopic perforation treatment [24]. In our study, successful endoscopic closure was possible for 39.3% (22/56) of ICPs. In fact, endoscopic treatment was tried in 25 cases with ICPs, but 3 cases needed operative treatment because of endoscopic treatment failure. Therefore, endoscopic repair of ICPs was successful in 88.0% (22/25).

Regardless of type of ICP, the patients with successful endoscopic repair were mostly females (68.2% vs. 32.4%, *p* = 0.009) and had smaller median size of perforation (0.81 vs. 1.30 cm, *p* = 0.001), more frequent immediate detection (86.4 vs. 26.5%, *p* = 0.000), and shorter hospital stay (8.6 vs. 15.8 days, *p* = 0.007) compared to the surgical repair group (Table 3). These findings suggest that endoscopic treatment can be applied to immediately detected, small ICPs. Especially, the time of detection is a critical factor, since the patients diagnosed after 24 h following colonoscopy have a higher rate of fecal contamination. Immediate detection of perforation makes endoscopic treatment possible and results in a shorter hospital stay. In addition, the perforation size is also important as found in our results. All 16 cases with perforation 1 cm or smaller in diameter among 33 patients with DICP were eligible for endoscopic repair, while the other 17 with DICP over 1 cm were managed surgically (Table 3).

There was no statistical difference in the fasting period between the surgical and the endoscopic treatment groups. However, surgery is associated with significant morbidity and mortality. Patients with ICP could have a high morbidity and mortality rate depending on the patient’s medical conditions, nature of the perforation, and methods of colonic perforation management. The 30-day morbidity and mortality rates are 21–53 and 0–26%, retrospectively [2, 25, 26]. The average length of hospital stay with colonoscopy perforation is 1–3 weeks [5, 26, 27]. The current study showed a mortality of 7.1%, and most fatalities were found to have serious comorbidities such as cerebrovascular accident, liver cirrhosis, and chronic obstructive pulmonary disease (Table 4). Surgical infection is the most common complication, while cardiopulmonary complications and multiple-organ failure are the leading causes of death [5, 6]. In our study, patients also died of multiple-organ failure or sepsis due to postoperative infection.

There were several limitations to our study. First, data were collected retrospectively, which is an important aspect in deciding between endoscopic treatment and surgery. This was not investigated thoroughly because of the retrospective design. Therefore, prospectively randomized-controlled data are needed to establish management strategies with strong evidence. Second, despite the large number of colonoscopies investigated, the number of enrolled patients was relatively small and did not allow us to show statistical significance in clinical outcome according to management of perforation, and in the clinical outcome according to the purpose of colonoscopy.

In conclusion, DICP was most frequent in the rectosigmoid area and in older women, and it could be detected immediately. Immediate detection and small size of perforation could be important factors for successful endoscopic repair, regardless of type of ICP. Careful attention and gentle manipulation in colonoscopy are needed to avoid the development of ICP or to detect it early, especially at the time of passing through rectosigmoid area of older women.

## References

[1] Wullstein C, Koppen M, Gross E (1999). Laparoscopic treatment of colonic perforations related to colonoscopy. Surg Endosc.

[2] Luning TH, Keemers-Gels ME, Barendregt WB, Tan AC, Rosman C (2007). Colonoscopic perforations: a review of 30,366 patients. Surg Endosc.

[3] Carpio G, Albu E, Gumbs MA, Gerst PH (1989). Management of colonic perforation after colonoscopy: report of three cases. Dis Colon Rectum.

[4] Cha JM (2014). Colonoscopy quality is the answer for the emerging issue of interval cancer. Intest Res.

[5] Iqbal CW, Cullinane DC, Schiller HJ, Sawyer MD, Zietlow SP, Farley DR (2008). Surgical management and outcomes of 165 colonoscopic perforations from a single institution. Arch Surg.

[6] Lohsiriwat V, Sujarittanakarn S, Akaraviputh T, Lertakyamanee N, Lohsiriwat D, Kachinthorn U (2008). Colonoscopic perforation: a report from World Gastroenterology Organization endoscopy training center in Thailand. World J Gastroenterol.

[7] Korman LY, Overholt BF, Box T, Winker CK (2003). Perforation during colonoscopy in endoscopic ambulatory surgical centers. Gastrointest Endosc.

[8] Baillie J (1994). Complications of endoscopy. Endoscopy.

[9] Panteris V, Haringsma J, Kuipers EJ (2009). Colonoscopy perforation rate, mechanisms and outcome: from diagnostic to therapeutic colonoscopy. Endoscopy.

[10] Dafnis G, Ekbom A, Pahlman L (2001). Complications of diagnostic and therapeutic colonoscopy within a defined population in Sweden. Gastrointest Endosc.

[11] Iqbal CW, Chun YS, Farley DR (2005). Colonoscopic perforations: a retrospective review. J Gastrointest Surg.

[12] Wess L, Eastwood MA, Wess TJ, Busuttil A, Miller A (1995). Cross linking of collagen is increased in colonic diverticulosis. Gut.

[13] Hollander E, David G (1993). Inverted sigmoid diverticulum simulating polyps. Orv Hetil.

[14] Damore LJ, Rantis PC, Vernava AM, Longo WE (1996). Colonoscopic perforations: etiology, diagnosis, and management. Dis Colon Rectum.

[15] Haubrich WS, Haubrich WS, Schaffner F, Berk JE (1995). Anatomy of the colon. Bockus gastroenterology.

[16] Kim JS, Kim BW, Kim JI (2013). Endoscopic clip closure versus surgery for the treatment of iatrogenic colon perforations developed during diagnostic colonoscopy: a review of 115,285 patients. Surg Endosc.

[17] Araghizadeh FY, Timmcke AE, Opelka FG, Hicks TC, Beck DE (2001). Colonoscopic perforations. Dis Colon Rectum.

[18] Farley DR, Bannon MP, Zietlow SP, Pemberton JH, Ilstrup DM, Larson DR (1997). Management of colonoscopic perforations. Mayo Clin Proc.

[19] Christie JP, Marrazzo J (1991). “Mini-perforation” of the colon–not all postpolypectomy perforations require laparotomy. Dis Colon Rectum.

[20] Hall C, Dorricott NJ, Donovan IA, Neoptolemos JP (1991). Colon perforation during colonoscopy: surgical versus conservative management. Br J Surg.

[21] Nelson RL, Abcarian H, Prasad ML (1982). Iatrogenic perforation of the colon and rectum. Dis Colon Rectum.

[22] Jentschura D, Raute M, Winter J, Henkel T, Kraus M, Manegold BC (1994). Complications in endoscopy of the lower gastrointestinal tract: therapy prognosis. Surg Endosc.

[23] Jung Y (2020). Endoscopic management of iatrogenic colon perforation. Clin Endosc.

[24] Taku K, Sano Y, Fu KI (2007). Iatrogenic perforation associated with therapeutic colonoscopy: a multicenter study in Japan. J Gastroenterol Hepatol.

[25] Cobb WS, Heniford BT, Sigmon LB (2004). Colonoscopic perforations: incidence, management, and outcomes. Am Surg.

[26] Teoh AY, Poon CM, Lee JF (2009). Outcomes and predictors of mortality and stoma formation in surgical management of colonoscopic perforations: a multicenter review. Arch Surg.

[27] Mai CM, Wen CC, Wen SH (2010). Iatrogenic colonic perforation by colonoscopy: a fatal complication for patients with a high anesthetic risk. Int J Colorectal Dis.

